# Epidermolysis Bullosa With Concomitant Amelogenesis Imperfecta: Clinical and Oral Implications in a Pediatric Patient

**DOI:** 10.7759/cureus.117553

**Published:** 2026-10-06

**Authors:** Wissal Kabbassi, Hind Ramdi, Mustapha El Alloussi

**Affiliations:** 1 Pediatric Dentistry, Faculty of Dental Medicine, Mohammed V University of Rabat, Rabat, MAR; 2 Pedodontics and Preventive Dentistry, Center for Research in Health Sciences (CRESS), Faculty of Dentistry, International University of Rabat, Rabat, MAR

**Keywords:** amelogenesis imperfecta, case report, epidermolysis bullosa, oral manifestations, pediatric dentistry

## Abstract

Epidermolysis bullosa (EB) is a rare inherited mechanobullous disorder characterized by extreme fragility of the skin and mucous membranes, leading to blister formation following minimal trauma. Among its different subtypes, the junctional form is frequently associated with dental abnormalities, particularly defects in enamel formation. Amelogenesis imperfecta (AI) is a hereditary enamel disorder affecting both primary and permanent dentitions and may present as quantitative or qualitative enamel defects. The coexistence of EB and AI reflects a shared molecular basis involving mutations in genes essential for epithelial adhesion and enamel development. This article reports a case of AI associated with EB in a child and emphasizes, through a review of the literature, the genetic relationships underlying the link between these two conditions.

## Introduction

Hereditary epidermolysis bullosa (HEB) comprises a heterogeneous group of rare, inherited genetic disorders characterized by marked fragility of the skin and/or mucous membranes, resulting in localized or generalized blistering following minimal or even no apparent trauma. These conditions arise from mutations in genes encoding structural proteins essential for the integrity and functional stability of the dermal-epidermal junction [1].

Epidemiological data on the incidence and prevalence of epidermolysis bullosa (EB) vary considerably among studies. Epidemiological estimates of inherited EB vary across studies. In a 16-year U.S. registry study including 3,271 patients, the overall incidence and prevalence of inherited EB were estimated at 19.57 and 11.07 cases per million, respectively [2,3].

Based on the ultrastructural level of skin cleavage, from the superficial to the deeper layers, EB is classified into distinct subtypes: EB simplex (EBS), junctional EB (JEB), dystrophic EB (DEB), and Kindler’s EB (KEB). A minimum of 20 causative genes have been identified.

Due to the ectodermal origin of the oral epithelium, including the gingiva, oral mucosa, and dental enamel, the oral cavity is frequently affected in EB. Furthermore, the oral environment, characterized by a high microbial load, predisposes patients to both primary disease manifestations and secondary chronic infections.

Mutations in specific genes, particularly those associated with JEB, also lead to abnormalities in dental enamel development, representing a syndromic amelogenesis imperfecta (AI) [4,5]. The aim of this article is to highlight the association between HEB and AI, and to present a clinical case illustrating their coexistence.

## Case presentation

An 11-year-old male patient presented for dental care due to carious lesions and a long-standing complaint of tooth discoloration. His dental history revealed similar findings in the primary dentition, characterized by early loss of tooth structure. The patient was born to consanguineous parents, with no similar condition reported among his siblings.

The medical history indicated that, at birth, the child exhibited a cutaneous disorder characterized by skin eruptions affecting the limbs and the diaper area. He was born at full term. A skin biopsy performed at that time confirmed the diagnosis of EB.

Cutaneous examination of the face revealed diffuse xerosis, predominantly affecting the perioral and mentonian regions. The skin appeared dry, thin, and fragile, with fine superficial scaling, particularly around the lips and chin. The epidermis showed a slightly atrophic and delicate texture, suggestive of reduced cutaneous resilience (Figure 1).

**Figure 1:**
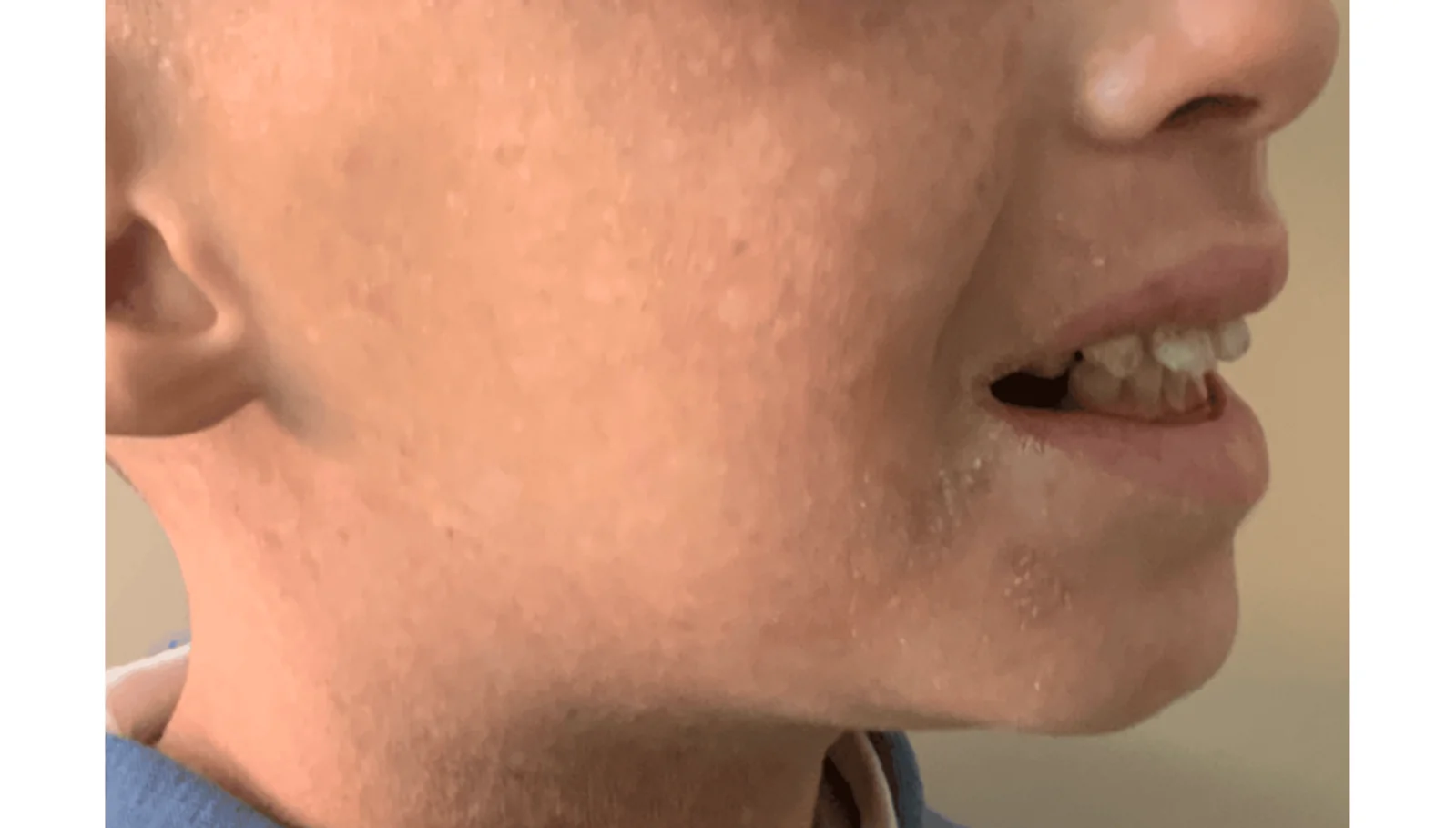
Skin view of the face

Examination of the hands revealed marked cutaneous abnormalities. The dorsal aspects showed abnormally thin, dry, and atrophic skin with a wrinkled and fragile appearance, associated with diffuse erythema, more pronounced on the left hand, and small cicatricial lesions. The fingers appeared elongated, and the translucent skin allowed partial visualization of the underlying structures. On the palmar aspects, the skin appeared thin and dry with a smooth, slightly shiny surface and reduced dermatoglyphic markings. Mild erythema and superficial fissuring were observed (Figures 2, 3).

**Figure 2:**
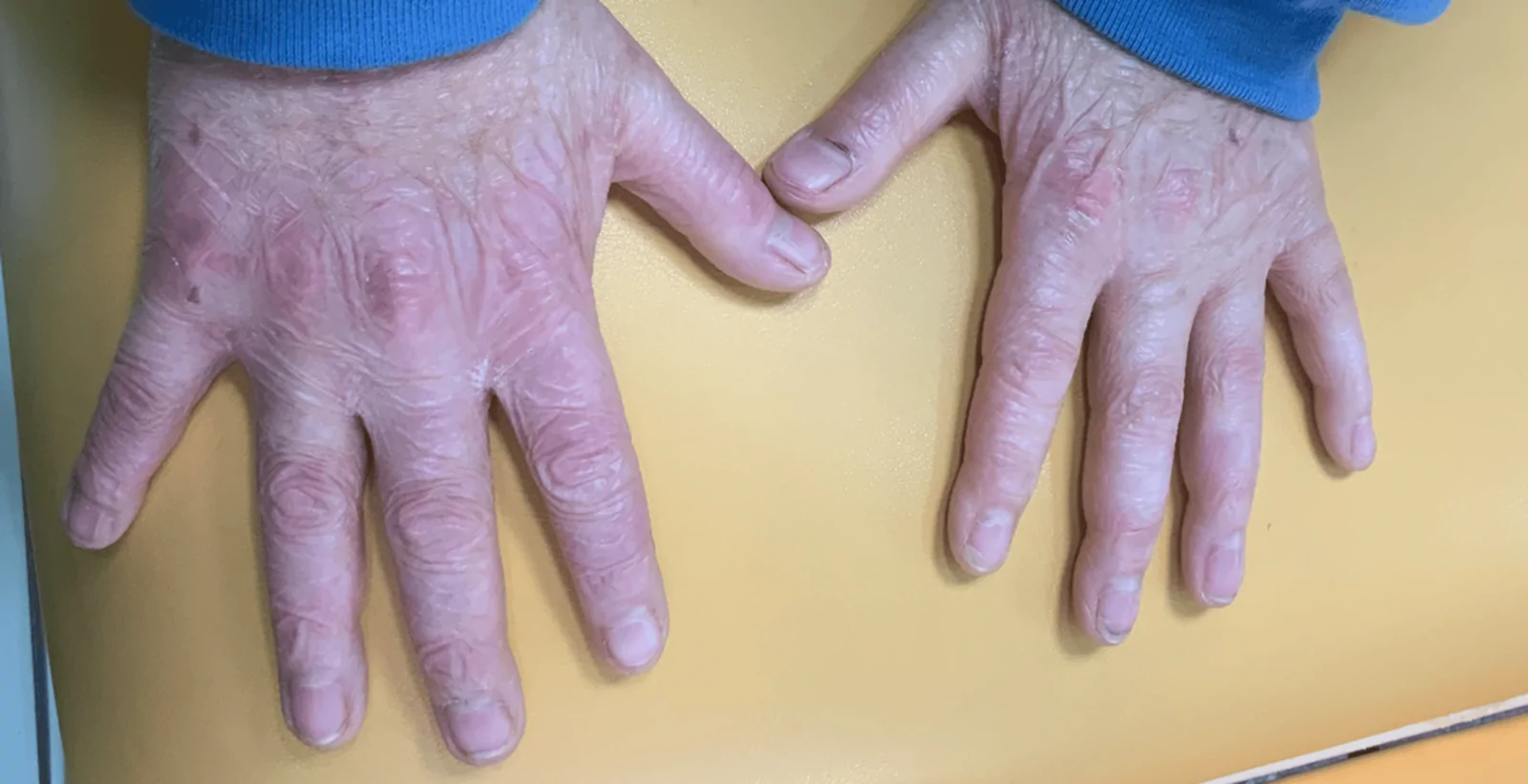
View of the dorsal surface of the hands

**Figure 3:**
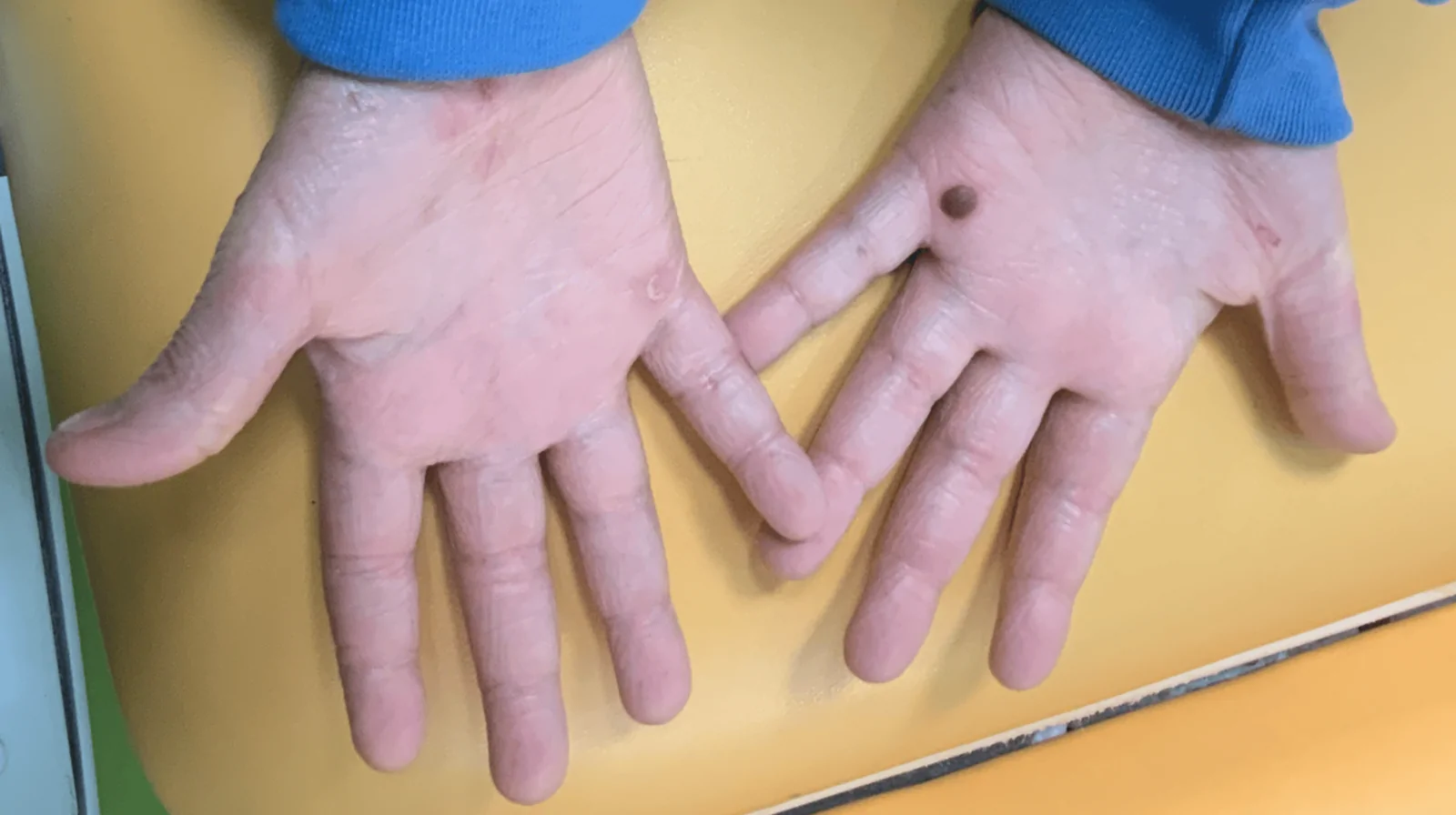
View of the palmar surface of the hands

Intraoral examination revealed generalized enamel abnormalities affecting both primary and permanent dentitions. The enamel was of normal or near-normal thickness, but appeared opaque, chalky, and yellowish-white, with loss of translucency. The surface was irregular and rough, showing areas of post-eruptive breakdown and pitting, particularly on occlusal and cervical surfaces. Brownish discolorations were observed, likely related to secondary staining. Localized gingival inflammation was present, presumably due to increased plaque retention associated with the defective enamel surface (Figure 4).

**Figure 4:**
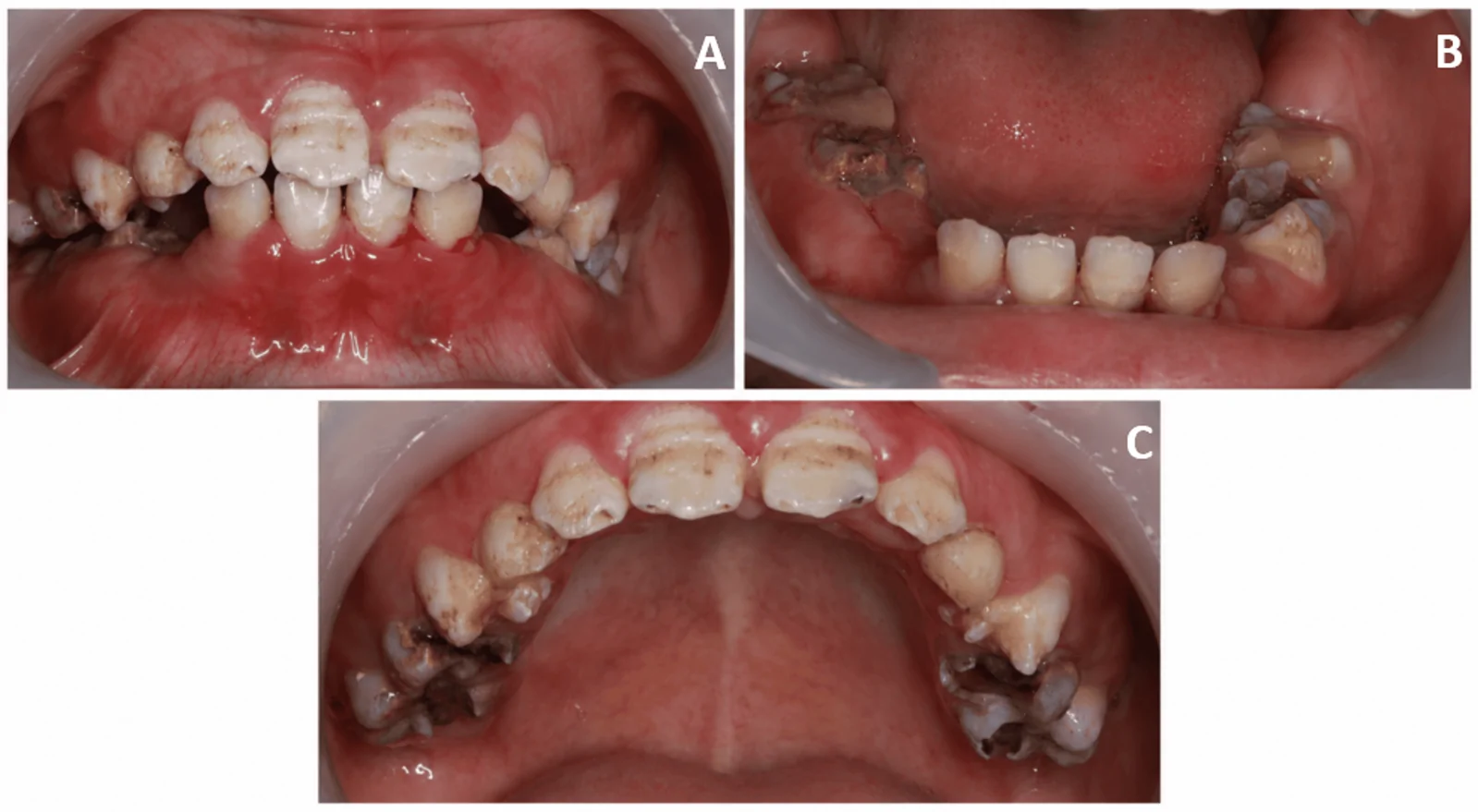
(A-C) Intraoral clinical photographs showing generalized enamel defects and severe dental caries

Panoramic radiography demonstrated an enamel layer of comparable thickness to normal enamel, but with reduced radiodensity, showing only slightly higher than the underlying dentin. The enamel-dentin junction was poorly defined in several teeth. No significant abnormalities in root development were observed (Figure 5).

**Figure 5:**
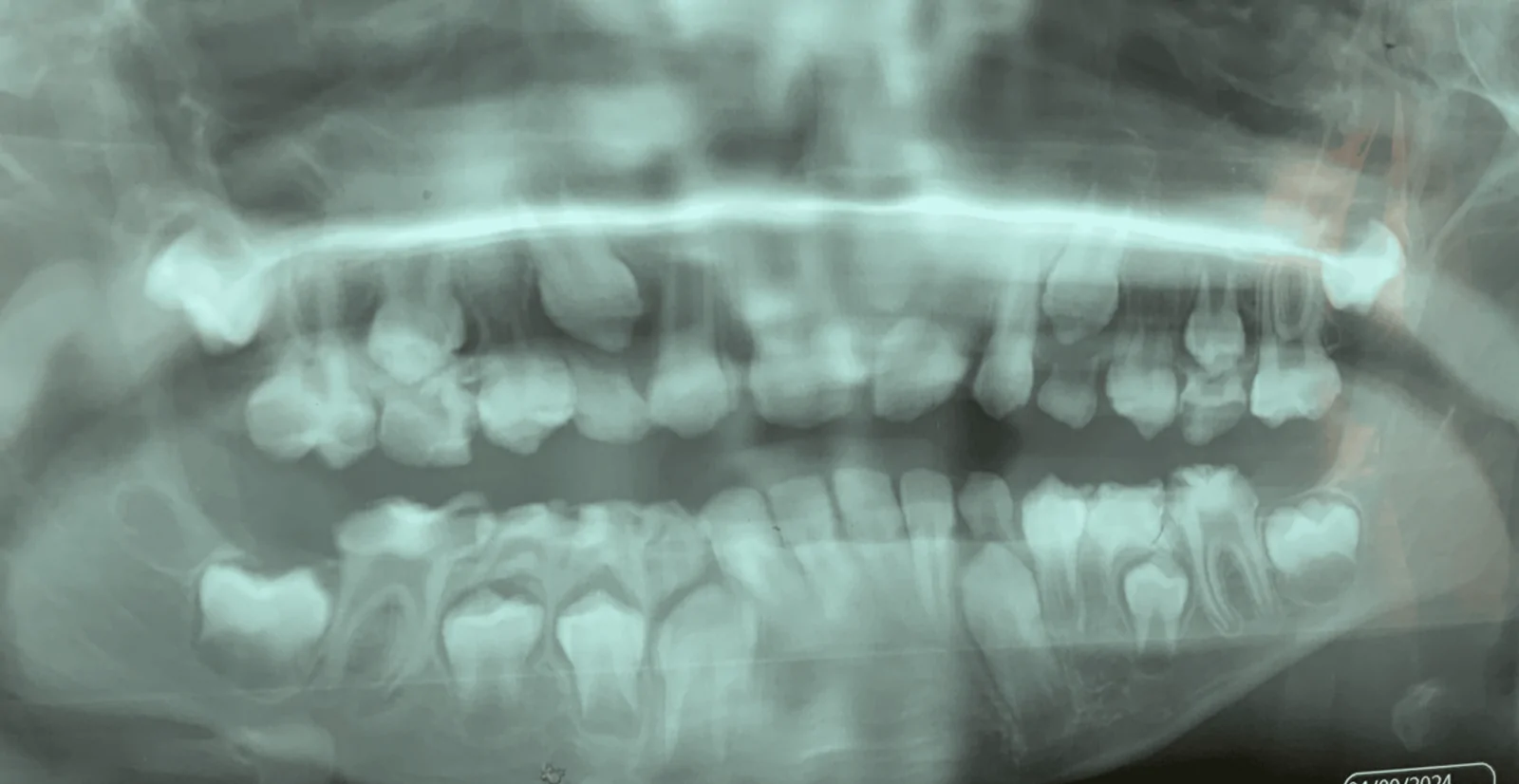
Preoperative panoramic X-ray

Taken together, the clinical and radiographic features are consistent with hypomaturation-type AI, characterized by defective enamel maturation leading to structurally fragile enamel that undergoes post-eruptive deterioration [6]. Initial periodontal therapy and oral hygiene reinforcement were essential to establish a healthy oral environment before restorative procedures. Stainless steel crowns were selected for severely compromised permanent molars because they provide durable full-coronal coverage and effectively protect structurally weakened teeth. Direct composite veneers were subsequently placed on the maxillary incisors to restore anterior esthetics while preserving as much tooth structure as possible.

## Discussion

Inherited EB comprises a heterogeneous group of rare genetic disorders characterized by skin and mucosal fragility resulting from mutations affecting proteins responsible for dermal-epidermal adhesion. According to the level of tissue cleavage, EB is classified into four major types: EBS, JEB, DEB, and Kindler’s syndrome. Table 1 summarizes the types of EB with the responsible genes [1].

**Table 1:** Classification of four major epidermolysis bullosa (EB) types [1] HEB: hereditary epidermolysis bullosa

HEB type	Skin cleavage	Inheritance	Gene affected
EB simplex (EBS)	Intraepidermal	Autosomal dominant, autosomal recessive	KRT5/KRT14/KLHL24/PLEC, PLEC/KRT5/KRT14/DST/EXPH5/CD151
Junctional EB (JEB)	Intralamina lucida	Autosomal recessive	LAMA3/LAMB3 LAMC2/COL17A/ITGA6/ITGB4/ITGB4/ITGA3
Dystrophic (DEB)	Sublamina densa	Autosomal dominant, autosomal recessive	COL7A1, COL7A1
Kindler’s syndrome (KEB)	Variable and mixed	Autosomal recessive	FERMT1

Among these subtypes, JEB is the form most consistently associated with developmental enamel defects because several of the causative genes are also involved in amelogenesis. Consequently, oral manifestations represent an integral component of the disease and may considerably affect patients' quality of life [1,2].

AI is a clinically and genetically heterogeneous group of inherited enamel defects affecting both the primary and permanent dentitions. AI may occur as an isolated condition or as part of a syndromic disorder. The association between EB and AI has been well documented, particularly in patients with JEB, reflecting a shared molecular basis involving genes that participate in both epithelial adhesion and enamel formation [3,4].

Several studies have demonstrated that pathogenic variants in LAMA3, LAMB3, LAMC2, and COL17A1 are responsible for many forms of JEB associated with AI. These genes encode structural components of the hemidesmosomal complex, including laminin-332 and type XVII collagen, which are essential for maintaining epithelial integrity. During tooth development, these proteins are also expressed at the epithelial-mesenchymal interface and contribute to normal ameloblast differentiation and enamel formation. Their disruption results in defective enamel maturation, explaining the generalized enamel abnormalities frequently observed in patients with JEB-associated AI [3,5-7].

In the present case, the patient presented with generalized hypomaturation-type AI associated with HEB diagnosed by skin biopsy during infancy. Although molecular genetic testing was not available, the coexistence of EB and generalized hypomaturation AI strongly suggests a junctional subtype, as previously described in patients carrying pathogenic variants in LAMA3, LAMB3, LAMC2, or COL17A1. Nevertheless, this interpretation should be made with caution because the underlying genetic mutation could not be confirmed.

Delayed diagnosis and the absence of early preventive dental care may have contributed to extensive enamel breakdown, multiple carious lesions, pulpal involvement, and premature loss of several teeth in our patient. These findings illustrate the rapid progression of oral disease when enamel defects remain untreated. The defective enamel surface promotes plaque accumulation, increases susceptibility to hypersensitivity and caries, and significantly compromises mastication, esthetics, and oral health-related quality of life.

The therapeutic approach in our case was guided by the need to preserve the remaining dentition, restore oral function, and improve esthetics while minimizing further loss of tooth structure. Given the generalized enamel defects and the structural vulnerability of the affected teeth, treatment planning required a balance between the durability of restorations and the preservation of healthy dental tissues.

The initial emphasis on periodontal health and oral hygiene was particularly important in establishing a favorable oral environment before restorative treatment. In patients with generalized enamel defects, effective plaque control and preventive care are essential to reduce the risk of caries and periodontal complications and to improve the longevity of subsequent restorations.

For severely compromised permanent molars, full-coronal coverage was considered appropriate because these teeth presented substantial structural loss and required protection against further fracture and wear. Stainless steel crowns offer a conservative and durable restorative option for posterior teeth in children, particularly when the remaining tooth structure is insufficient to support conventional direct restorations.

For the maxillary anterior teeth, direct composite restorations provided an esthetic solution while allowing maximal preservation of tooth structure. This approach is particularly relevant in growing patients, in whom extensive prosthetic rehabilitation may be deferred until craniofacial and dental development is more advanced. Moreover, the relative ease of repair or modification of direct composite restorations can be advantageous during growth.

Management of patients with EB requires special precautions because mucosal fragility increases the risk of soft tissue injury during routine dental procedures. Care should therefore emphasize gentle manipulation of oral tissues, adequate lubrication of instruments, and preventive strategies aimed at minimizing the need for invasive treatment. In severe cases, treatment under general anesthesia may be necessary; however, careful preoperative assessment is essential because airway involvement and mucosal fragility may complicate anesthetic management [8-10].

A limitation of this case report is that parental and sibling dental and dermatological examinations were not performed, and genetic counseling was not undertaken. Therefore, the familial clinical phenotype and inheritance pattern could not be further characterized. In addition, molecular genetic testing was not available for the patient, precluding confirmation of the specific EB subtype and the underlying mutation.

This case highlights the importance of early dental assessment in children diagnosed with EB. Close collaboration between dermatologists, pediatricians, geneticists, and pediatric dentists is essential for early recognition of enamel defects, implementation of preventive measures, and timely restorative management. Such multidisciplinary care can substantially reduce oral complications, preserve dental function, and improve the long-term quality of life of affected children.

## Conclusions

The oral cavity may be affected by multiple complications in HEB, with their nature and severity varying according to the clinical subtype and disease severity. The link between HEB, particularly JEB, and AI has been genetically established and reported by studies. Early recognition of dental manifestations in patients with EB is essential to ensure timely diagnosis and appropriate multidisciplinary management. Increased awareness among healthcare professionals may facilitate early referral, optimize preventive and restorative care, and ultimately reduce the functional, aesthetic, and psychosocial burden associated with these conditions.
